# Sirtuin1 meditated modification of Notch1 intracellular domain regulates nucleolar localization and activation of distinct signaling cascades

**DOI:** 10.3389/fcell.2022.988816

**Published:** 2022-09-23

**Authors:** Neetu Saini, Geetha Bheeshmachar, Apurva Sarin

**Affiliations:** Regulation of Cell Fate, Institute for Stem Cell Science and Regenerative Medicine (DBT-inStem), Bengaluru, Karnataka, India

**Keywords:** NOTCH1, Sirtuin1, nucleolus, acetylation, DNA damage, apoptosis

## Abstract

Notch signaling is involved in cell fate decisions in the development and maintenance of tissue homeostasis. Spatial regulation of the Notch1 intracellular domain (NIC1), has been shown to underpin signaling outcomes mediated by this receptor. We recently reported a putative Nucleolar Localization Sequence (NoLS) in NIC1. Here we investigate if the putative NoLS identified in NIC1 regulates localization in the nucleolus and anti-apoptotic activity. Confocal imaging of live cells expressing NIC1 or forms modified by deletion or site-directed mutagenesis established that the putative NoLS in NIC1 is required for nucleolar localization and regulated by the deacetylase Sirtuin1. Subsequent analysis of anti-apoptotic activity revealed signaling cascades linked to nucleolar localization. For this, etoposide and 4-Nitroquinoline 1-oxide, an inhibitor of topoisomerase-II and a UV mimetic drug respectively, were used as prototypic triggers of genomic damage in a mammalian cell line. While NIC1 blocked apoptosis regardless of its localization to the nucleoplasm or nucleolus, modifications of NIC1 which promoted localization to the nucleolus triggered a dependence on the nucleolar proteins fibrillarin and nucleolin for anti-apoptotic activity. Further, cells co-expressing NIC1 and Sirtuin1 (but not its catalytically inactive form), confirmed both spatial regulation and the switch to dependence on the nucleolar proteins. Finally, site-directed mutagenesis showed that the NoLS lysine residues are targets of Sirtuin1 activity. NIC1 mediated transcription is not similarly regulated. Thus, NIC1 localization to the nucleolus is regulated by Sirtuin1 modification of the lysine residues in NoLS and triggers a distinct signaling cascade involving nucleolar intermediates for anti-apoptotic activity.

## Introduction

Post-translational modifications reversibly tune protein stability, localization, and interaction with other proteins (Pawson and Scott 2005; Yang and Seto, 2008; Deribe et al., 2010; Bauer et al., 2015; Shimizu et al., 2021) and are frequently observed to regulate signaling pathways. Notch signaling is an evolutionarily conserved signaling pathway shown to be regulated by reversible post-translational modifications at multiple steps of the core pathway (Antfolk et al., 2019). Notch signaling is typically activated by the binding of single-pass membrane receptor to its ligands in a juxtacrine manner. Ligand interaction induces two successive proteolytic cleavages by ADAM10/TACE and γ-secretase in the receptor resulting in the release of Notch intracellular domain (NIC) (Kovall et al., 2017). NIC then translocates to the nucleus where, in mammals, it associates with CSL family protein-RBP-jk, mastermind-like protein (MAML), and other co-activators-p300 and PCAF, to activate downstream genes transcription of Hes and Hey family (Wallberg et al., 2002; Kopan and Ilagan, 2009; Kovall et al., 2017). Four Notch receptors and five ligands namely, Notch1-Notch4, and Jagged (Jag1, Jag2) and Delta-like (Dll1, Dll3 and Dll4) respectively are shown to be expressed in mammals (Dumortier et al., 2005; D’Souza et al., 2008).

Notch receptors are modified by acetylation, fucosylation, phosphorylation, methylation, hydroxylation, and sumoylation (Coleman et al., 2007; Fortini 2009; Ishitani et al., 2010; Guarani et al., 2011; Popko-Scibor et al., 2011; Ranganathan et al., 2011; Antila et al., 2018). Post-translational modifications of the receptors add to the diversity of signaling outcomes linked to the activation of the Notch pathway. Thus, fucosylation of Notch1 extracellular domain by *O*-fucosyltransferase 1 (Pofut1) is required for interaction with the ligand (Okajima & Irvine 2002) and acetylation, phosphorylation and methylation of NIC regulate stability and turnover of the protein (Popko-Scibor et al., 2011; Hein et al., 2015; Lee et al., 2015). Acetylation and phosphorylation of NIC are also shown to regulate intracellular localization of NIC (Ramakrishnan et al., 2015; Marcel et al., 2017). Akt-dependent phosphorylation of NIC4 at S1495, S1847, S1865 and S1917 promotes interaction with 14-3-3 which in turn cloistered NIC4 in the cytoplasm (Ramakrishnan et al., 2015). PI3K/AKT mediated phosphorylation of NIC1 sequesters NIC1 in the cytoplasm (Baek et al., 2007; Song et al., 2008). Acetylation of NIC1 specifically at K2157, K2160, K2164, and K2174 enforces NIC1 localization in the nucleus in regulatory-T cells (Marcel et al., 2017). In addition to being part of NIC1 transcriptional complex, p300 and PCAF are also implicated in the acetylation of 16 lysine residues spanning across the NIC1 (Guarani et al., 2011; Popko-Scibor et al., 2011). NAD + dependent deacetylase-Sirtuin1 removes acetyl group from the lysine residues in NIC1 (Guarani et al., 2011; Popko-Scibor et al., 2011). Acetylation of distinct lysine residues leads to a different regulation on NIC1 such as stability or localization, however, the understanding of acetylation mediated modification of NIC1 functions is incomplete.

Although in-silico analysis of Notch protein sequence indicated a putative Nucleolar Localization Sequence (NoLS) in many Notch proteins in different species, only a few studies have reported Notch signaling activated from the nucleolus (Sarikaya and Jerome-Majewska, 2011; Tan et al., 2014; Saini & Sarin, 2020). The NoLS in NIC1 (in different species), is enriched in lysine residues that are post-translationally modified by sumoylation and acetylation (Guarani et al., 2011; Antila et al., 2018; Saini & Sarin 2021). Hence, we test the hypothesis that post-translational modification of lysine residues in the putative NoLS in NIC1 regulates its localization in the nucleolus. The functional consequences of this are assessed through NIC-mediated inhibition of apoptosis induced by an ionization radiation mimetic or a genotoxic drug. We provide evidence that post-translational modification of lysine residues plays a key role in nucleolar localization of NIC1. Further, we present evidence that nucleolar localized NIC1, adapts to interact with local proteins to activate distinct signaling cascades conferring protection from apoptosis triggered by genomic damage. Evidence that transcriptional outcomes of NIC1 signaling may be uncoupled from protection from genomic damage, is also presented.

## Materials and methods

### Cells

HEK293T (HEK) cell line was obtained from American Type Culture Collection (ATCC) (Manassas, VA, USA) and maintained in Dulbecco’s Modified Eagle’s Medium (DMEM) (GIBCO, Life Technologies, Carlsbad, CA, USA) supplemented with 0.1% penicillin/streptomycin and 10% heat-inactivated fetal bovine serum (FBS) (Scientific Hyclone TM, Waltham, MA, USA)-(DMEM-CM) at 37°C with 5% CO_2_. HEK293T cells till passage 25 were used for experiments. *Mycoplasma* contamination in the cultures was routinely tested using the MycoAlertTM *Mycoplasma* Detection Kit (Lonza, Basel, Switzerland).

### Chemicals and antibodies

Thapsigargin (TG, T9033) and 4NQO (N8141) were purchased from Sigma-Aldrich (St. Louis, MO, USA). Etoposide (341205) was from Calbiochem-Merck Millipore (Darmstadt, Germany). Dharmafect1 and siRNA to Fibrillarin (L-011269), Nucleolin (L-003854) and scrambled control (D-0018010) were from Dharmacon (Lafayette, CO, USA). Antibody to Sirtuin1 (9475) was from Cell Signaling Technology (MA, USA). Antibody to Tubulin (MS-581-P0) was from Neomarker (Fremont, CA, USA). Trizol (15596026) and SYBR™ Green Master Mix were from Thermo Scientific (Waltham, MA, USA). PrimeScript 1st strand cDNA Synthesis Kit (6110A) was purchased from Takara Bio (Shiga, Japan).

### Plasmids

Sirt1 and Sirt1H363Y were a gift from Michael Greenberg (Addgene plasmid# 1791 and 1792; http://n2t.net/addgene:1791; RRID: Addgene_1791 and RRID: Addgene_1792). mTagRFP-T-Fibrillarin-7 was a gift from Michael Davidson (Addgene plasmid #58016). NIC1-GFP was genetrated in-house as described earlier (Saini et al., 2022). NIC1-NoLS 4KR-GFP, NIC1-NoLS 4KA-GFP, NIC1-ΔTADPEST-GFP and NIC1-ΔTADPEST 4KA-GFP were generated in-house using the following primers (5′-3′):

NIC1-NoLS 4KR-GFP Forward: GGT​TCC​CTG​AGG​GCT​TCC​GAG​TGT​CTG​AGG​CCA​GC CGGCGGCGGCGG CGGGAGCCCCTCGG

NIC1-NoLS 4KR-GFP Reverse: ATC​GAA​TTC​TAT​GCG​CAA​GCG​CCG​GCG​GCA​GCA​TG GCCAGCTCTGGTTC CCT​GAG​GGC​TTC​CGA​GTG​TC

NIC1-ΔTADPEST EcoR1 Forward: ACT​GAA​TTC​TAT​GCG​GCG​GCA​GCA​T

NIC1 -ΔTADPEST BamHI Reverse: AAT​GGA​TCC​CTT​GAA​GGC​CTC​CGG

NIC1-ΔTADPEST 4KA/ NIC1-NoLS 4KA Forward: GCA​GTG​TCT​GAG​GCC​AGC​GCG​GCG​GCG​CGG​CGG GAGCCCCTCGGCGAG

NIC1-ΔTADPEST 4KA/ NIC1-NoLS 4KA Reverse: CGC​CGC​CGC​GCT​GGC​CTC​AGA​CAC​TGC​GAA​GCC CTCAGGGAACCA GAGCTGGCC

Construct sequences were verified by automated Sanger sequencing conducted in-house.

### RT PCR analysis

0.5 × 10^6^ HEK cells were lysed in 1 ml of TRIzol and RNA isolation was performed according to the manufacturer’s instructions. 1 µg RNA was used for cDNA synthesis using PrimeScript 1st strand cDNA Synthesis Kit (Takara Bio). cDNA was diluted in 1:5 ratio and real-time PCR was performed using Maxima™ SYBR Green qPCR Master Mix and QuantStudio™ 5 Real-Time PCR System. Relative change in transcript levels was calculated using 2^–ΔΔCt^ method using glyceraldehyde 3-phosphate dehydrogenase (GAPDH) as a reference gene.

Primers used for RT PCR against Human genes: Forward (5′–3′); Reverse (5′–3′)

GAPDH: TGC​ACC​ACC​AAC​TGC​TTA​GC; GGC​ATG​GAC​TGT​GGT​CAT​GAG

Hes5: CCG​GTG​GTG​GAG​AAG​ATG​CG; GCG​ACG​AAG​GCT​TTG​CTG​TG

FBL: TGG​ACC​AGA​TCC​ACA​TCA​AA; GAC​TAG​ACC​ATC​CGG​ACC​AA

NCL: CCA​GCC​ATC​CAA​AAC​TCT​GT; TAA​CTA​TCC​TTG​CCC​GAA​CG

### Transfection

As described earlier in (Saini and Sarin, 2020), HEK cells at a density of 0.25 × 10^6^ were plated on the culture grade 35 mm dishes, 24 h post-plating cells were transfected with 100 nM siRNA using Dharmafect or plasmids at the indicated concentrations using lipofectamine 2000 as per the manufacturer’s instructions. To test the requirement of genes for NIC1 mediated anti-apoptotic activity, cells transfected with siRNA were cultured for 24–30 h and then harvested by trypsinization and re-plated at a density of 0.25 × 10^6^/35 mm dish. HEK cells treated with siRNA were transfected with the plasmids at the required concentrations using lipofectamine 2000 and cultured for 24 h before inducing the apoptotic damage. Plasmids were transfected at the following concentrations: pEGFP-C1 (1 µg), NIC1-GFP (2 µg), NIC1-ΔTADPEST-GFP (2 µg), NIC1-ΔTADPEST 4KA-GFP (2 µg), NIC1-NoLS 4KA-GFP (2 µg), NIC1-NoLS 4KR-GFP (2 µg), SIRT1 (3 µg) and SIRT1H363Y (3 µg) and Fibrillarin-RFP (1 µg). Total DNA transfected in the different transfection groups was equalized with pcDNA3.

### Analysis of apoptotic damage

HEK cells transfected with required plasmids were cultured for 24 h in DMEM-CM medium. The next day, cells were cultured in 2% FBS containing DMEM with or without etoposide (10 μM) or 4NQO (10 μM) for 48 h. To trigger apoptotic damage by thapsigargin, HEK cells transfected with NIC1-GFP, NIC1-ΔTADPEST 4KA-GFP or GFP were treated with thapsigargin (10 µM) for 24 h in serum-free DMEM. Cells were harvested and stained with Hoechst 33342 (1 μg/ml) as described in (Saini and Sarin, 2020) and nuclear morphology was assessed to score for nuclear damage in GFP-positive cells using fluorescent microscopy (Olympus BX-60). Samples were blinded for the experimenter and approximately 200 cells in 5-7 random fields were scored for apoptotic damage.

### Analysis of NIC1 localization

0.25 × 10^6^ HEK cells were plated onto sterile coverslips fixed in Petri dishes to allow for confocal imaging. 18–20 h post-plating, cells were co-transfected with NIC1 constructs tagged with GFP and Fibrillarin-RFP as described above using lipofectamine 2000 and cultured for another 24 h in DMEM-CM medium. The next day, cells were stained with Hoechst 33342 (1 μg/ml) as described in (Saini & Sarin 2020) and confocal images were acquired using Olympus FV3000 using 60X oil NA 1.35 oil-immersion objective. Images were processed and analyzed using Fiji-Image J software. To determine the fraction of NIC1 localized in the nucleolus, Mean Fluorescent Intensity (MFI) of different GFP (tagged to NIC1) in the nucleolus was measured by drawing ROI around the nucleolus based on the Fibrillarin localization in the nucleus. MFI of GFP (tagged to NIC1) in the nucleoplasm was measured by drawing ROI around the nucleolus based on the Fibrillarin localization and Hoechst staining in the nucleus. Fraction of NIC1 in the nucleolus=MFI of GFP in the nucleolus/MFI of GFP in the nucleoplasm.

### Western blotting

0.1 × 10^6^ HEK cells were lysed in 25 µl of SDS lysis buffer (2% SDS, 10% glycerol, 0.002% bromophenol blue, 200 mM DTT and 50 mM Tris-Cl pH 6.8) containing a protease inhibitor cocktail–aprotinin, leupeptin and pepstatin (2 μg/ml each), 1 mM PMSF, 1 mM NaF, 1 mM Na_3_VO_4_ and 10 μM MG132. The tube was vortexed for 20–30 s and incubated at 100°C for 10 min. Cell lysates were immediately resolved by SDS-PAGE and transferred to nitrocellulose membrane (GE Healthcare, Chicago, USA) and then blocked with 5% non-fat dried milk in Tris-buffered saline–Tween 20 (TBST) for 1 h at room temperature. Primary antibodies diluted in 5% non-fat dried milk in TBST at the following concentration: Sirtuin1 (1:500) and Tubulin (1:1000) were incubated overnight at 4°C. After incubation with primary antibodies, membranes were washed three times for 10 min each with TBST followed by incubation with horseradish peroxidase-conjugated secondary antibody (1:1000 dilutions) for 1 h at room temperature. After incubation with secondary antibody, membranes were washed three times with TBST. Membranes were developed using Super Signal West Dura substrate (Thermo Scientific, Waltham, MA, USA), and images were acquired using iBright FL1000 Invitrogen.

### Statistical analysis

Data are represented as mean ± standard deviation (Mean ± SD) derived from three independent experiments. Statistical significance was measured using unpaired student’s t-test and *p*-values ≤0.01 and ≤0.001 were considered statistically significant. *p*-value >0.05 were considered non-significant (ns).

## Results

### NIC1 signaling protects from genomic damage

**FIGURE 1:**
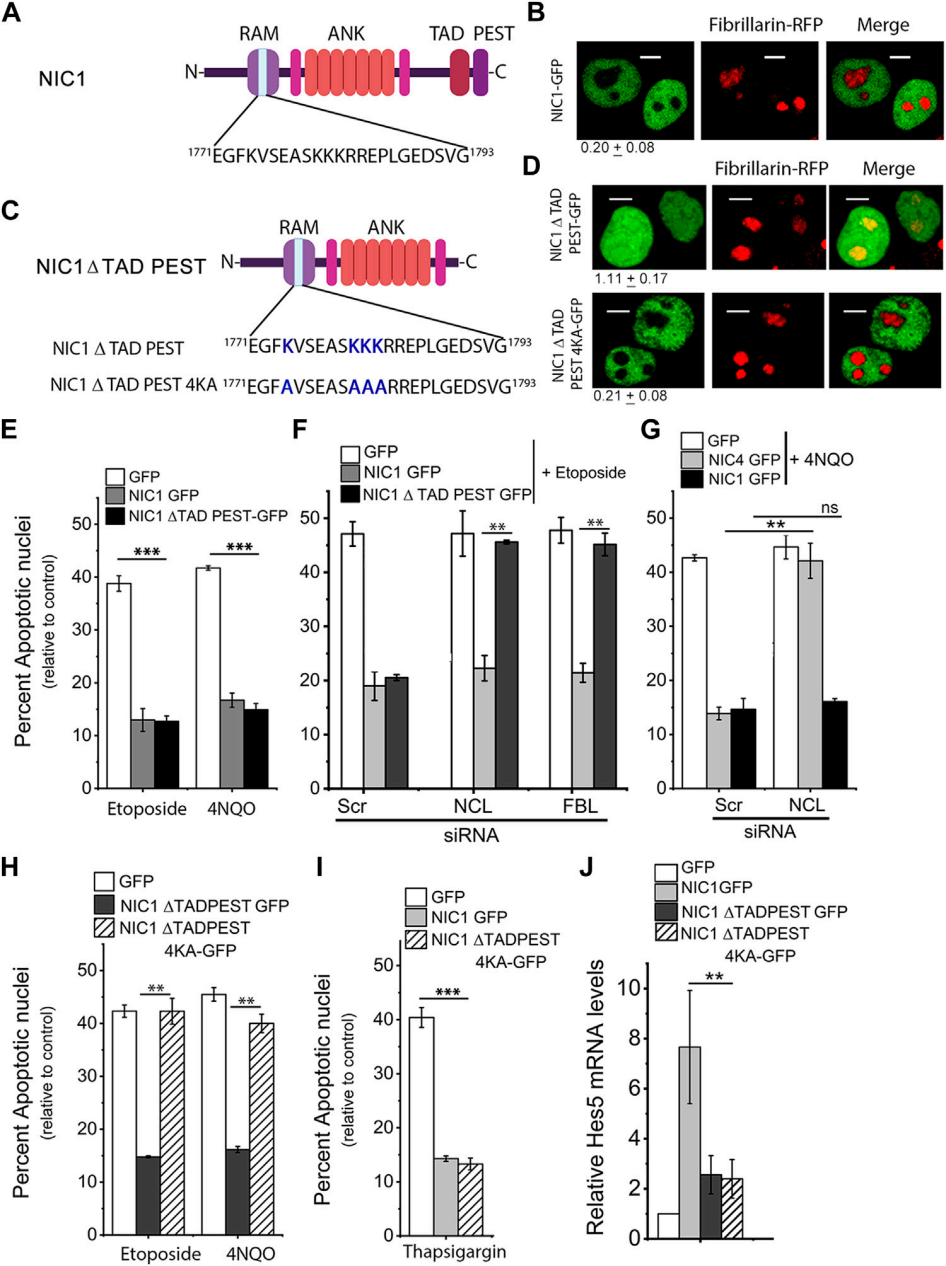
Deletion of TADPEST domains allows nucleolar localization of the Notch1 intracellular domain (NIC1). **(A)** Schematic showing the putative Nucleolar Localization Sequence (NoLS) in NIC1. **(B)** Representative confocal images of HEK cells co-expressing NIC1-GFP and Fibrillarin (FBL)-RFP imaged 24 h post-transfection. Images are representative of 70 cells across three independent experiments. Scale bar: 5 μm. 0.20 ± 0.08 shows the relative NIC1 levels in the nucleolus as compared to the nucleoplasm calculated as described in the methods. **(C)** Schematic to show the deletion of TAD and PEST domains in NIC1 and lysine (K) to alanine (A) mutation in the putative NoLS in NIC1-ΔTADPEST-GFP. **(D)** Representative confocal images of HEK cells co-transfected with NIC1-ΔTADPEST-GFP or NIC1-ΔTADPEST 4KA-GFP and Fibrillarin-RFP plasmids and cultured for 24 h in complete medium before imaging. Images are representative of at least 30 cells across two independent experiments. Scale bar: 5 μm. 1.11 ± 0.17 (NIC1 ΔTADPEST) and 0.21 ± 0.08 (NIC1-ΔTADPEST 4KA) show the relative NIC1 levels in the nucleolus as compared to the nucleoplasm. **(E)** Percent apoptotic nuclei in HEK cells expressing NIC1-ΔTADPEST-GFP, NIC1-GFP, or GFP cultured with etoposide (10 µM) or 4NQO (10 µM) for 48 h in medium containing 2% FBS and assessed for apoptotic damage as described in methods. **(F)** Percent apoptotic nuclei in HEK cells expressing NIC1-ΔTADPEST-GFP, NIC1-GFP, or GFP cultured with etoposide (10 µM) for 48 h in medium containing 2% FBS. HEK cells pre-treated with siRNA to Nucleolin (NCL), FBL, or scrambled control for 48 h were transfected with indicated plasmids, and cultured for 24 h in the complete medium, before etoposide treatment. **(G)** Percent apoptotic nuclei in HEK cells expressing NIC1-GFP, NIC4-GFP, or GFP cultured with 4NQO (10 µM) for 48 h in medium containing 2% FBS. HEK cells pre-treated with siRNA to NCL or scrambled control for 48 h were transfected with indicated plasmids, and cultured for 24 h in the complete medium, before 4NQO treatment. **(H)** Percent apoptotic nuclei in HEK cells expressing NIC1-ΔTADPEST-GFP, NIC1- ΔTADPEST 4KA-GFP or GFP cultured with etoposide (10 µM) or 4NQO (10 µM) for 48 h in medium containing 2% FBS. **(I)** Percent apoptotic nuclei in HEK cells expressing NIC1-ΔTADPEST-GFP, NIC1-GFP, or GFP cultured with Thapsigargin (10 µM) for 24 h in serum-free medium. **(J)** Relative *Hes5* mRNA levels in HEK cells transfected with NIC1-GFP, NIC1-ΔTADPEST-GFP, NIC1-ΔTADPEST 4KA-GFP or GFP and cultured in complete medium for 30 h. Data plotted as mean ± S.D. of three independent experiments. *** and ** show significant difference with *p*-value ≤ 0.001 and ≤0.01, respectively, and ns shows non-significant difference examined using the unpaired student’s t-test.

Analysis of NIC1 protein sequence using the **N**ucle**O**lar localization sequence **D**etector, (NoD, http://www.compbio.dundee.ac.uk/www-nod/) indicated a putative Nucleolar Localization Sequence (NoLS) in the NIC1, N-terminal (Figure 1A) (Saini and Sarin 2021). However, in our earlier work, which focused on the closely related protein Notch4, immuno-staining for Notch1 protein in Breast cancer cell lines-SUM149, Hs578T and HCC1806, showed that NIC1 was excluded from the nucleolus (Saini and Sarin 2020). Further, these data were recapitulated when localization of GFP tagged Notch1 intracellular domain (NIC1) expressed in HEK cells was examined. Thus, in live-cell confocal imaging of HEK cells co-transfected with the nucleolar protein Fibrillarin-RFP (FBL-RFP) (Aris & Blobel 1991; Shubina et al., 2016) NIC1-GFP was observed to localize in the nucleoplasm and was excluded from the nucleolus, marked by FBL-RFP (Figure 1B). FBL-RFP has been used a marker of the nucleolus in all experiments in this study. Relative NIC1 protein levels localized in the nucleolus were computed as described in the methods (Supplementary Figure S1A).

Post-translational modifications of Notch intracellular domains have been shown to regulate its subcellular localization (Ramakrishnan et al., 2015; Marcel et al., 2017). The putative NoLS in NIC1-GFP includes lysine residues (K1774, K1780, K1781 and K1782), which are modified by acetylation and sumoylation (Guarani et al., 2011; Antila et al., 2018). Acetylation of lysine residues neutralizes the positive charge and should abrogate NoLS function (Martin et al., 2015). Therefore, subsequent experiments tested the possibility that acetylation of NIC1 tunes localization to the nucleolus. Acetyltransferases, p300 and PCAF are reported to acetylate the lysine residues in NIC1 via interactions with the C-terminal domains of NIC1 (Kurooka and Honjo, 2000; Oswald et al., 2001; Guarani et al., 2011). Hence, the localization of a NIC1 deletion mutant that lacks the C-terminal Transactivation Domain (TAD) and PEST sequences (Figure 1C)–NIC1-ΔTADPEST–tagged to GFP, was assessed in cells co-expressing FBL-RFP. NIC1-ΔTADPEST-GFP was uniformly distributed in the nucleus with some co-localization with FBL-RFP (Figure 1D). To assess the requirement of the putative NoLS for localization of NIC1-ΔTADPEST-GFP, lysine residues K1774, K1780, K1781 and K1782 were changed to the neutral amino acid alanine (A) by site-directed mutagenesis (Figure 1C). Analysis of NIC1-ΔTADPEST 4KA-GFP localization in cells co-expressing FBL-RFP, showed that NIC1-ΔTADPEST 4KA-GFP was no longer detected in the nucleolus, although its localization to the nucleoplasm was unchanged (Figure 1D).

Nuclear localization of NIC1 has been shown to be critical for protection from genomic damage (Vermezovic et al., 2015). The Topoisomerase II inhibitor-etoposide and UV-mimetic drug 4-Nitroquinoline 1-oxide (4NQO) were used to induce genomic damage (Miao et al., 2006; Soubeyrand et al., 2010) and apoptotic damage scored in so treated HEK cells, expressing the different GFP tagged NIC1 recombinants relative to cells transfected with control vector. The analysis revealed striking differences between cells expressing NIC1-ΔTADPEST-GFP, compared to NIC1-GFP. NIC1-ΔTADPEST-GFP inhibited apoptosis induced by etoposide and 4NQO (Figure 1E). However, depletion of the nucleolar proteins NCL or FBL abrogated NIC1-ΔTADPEST-GFP mediated inhibition of apoptosis. In the same experiment, NIC1-GFP mediated anti-apoptotic activity was independent of NCL or FBL (Figure 1F). RNAi-mediated knockdown of NCL or FBL was assessed by their mRNA levels in siRNA treated groups as compared to the control scrambled group (Supplementary Figure S1B). For reference, we show data recapitulating published observations (Saini and Sarin 2020), where we reported a dependence on nucleolar proteins for NIC4 but not NIC1-mediated anti-apoptotic activity (Figure 1G and Supplementary Figure S1C). Intriguingly, the mutation of the lysine residues in the NoLS -NIC1-ΔTADPEST4KA-GFP- which was largely present in the nucleoplasm showed no inhibition of apoptosis induced by etoposide or 4NQO (Figure 1H). However, this was not attributable to loss of activity or stability of the modified protein, as NIC1-ΔTADPEST 4KA-GFP inhibited thapsigargin (an ER stressor)-induced apoptosis, to a level comparable to NIC1-GFP (Figure 1I). Transcriptional activity was assessed by mRNA induction of *Hes5*, a canonical target of Notch proteins, which was compromised in the NIC1 deletion mutant of the TAD-PEST domain (Figure 1J), as reported by others (Aster et al., 2000; Kurooka and Honjo, 2000; Kopan and Ilagan 2009).

The experiments that follow tested if acetylation of NIC1 regulates localization to the nucleolus. These experiments tested the effect of Sirtuin1 (Sirt1), an NAD^+^-dependent deacetylase, which is reported to deacetylate lysine residues including the lysine residues present in putative NoLS in NIC1 (Guarani et al., 2011).

### Sirt1 confers dependence on nucleolar proteins for NIC1 mediated anti-apoptotic activity

**FIGURE 2:**
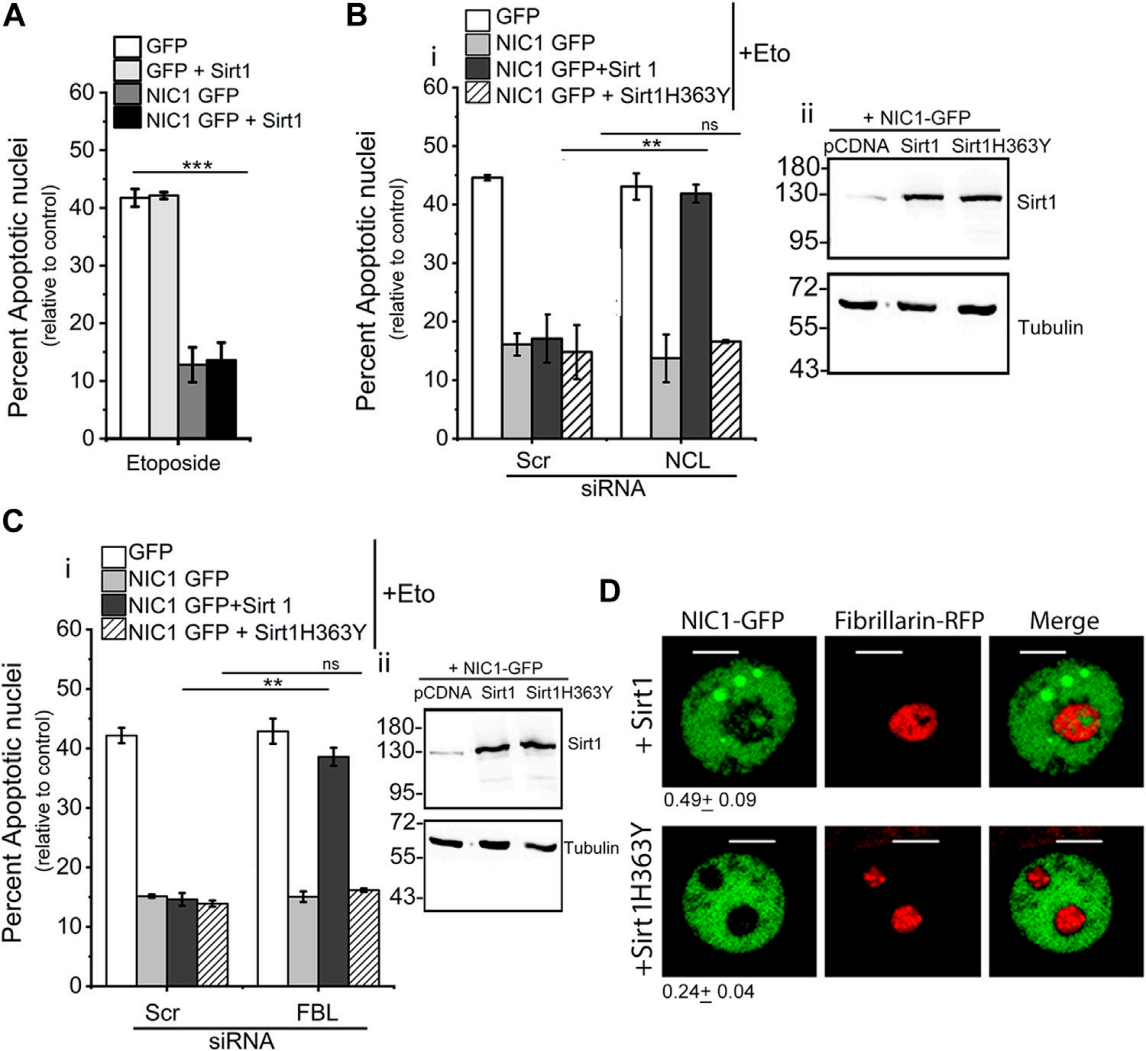
Co-expression of Sirt1 but not Sirt1H363Y with NIC1 promotes localization to the nucleolus. **(A)** Percent apoptotic nuclei in HEK cells expressing NIC1-GFP, NIC1-GFP + Sirtuin1 (Sirt1), GFP, or GFP + Sirt1 cultured with etoposide (10 µM) in medium containing 2% FBS for 48 h **(B,C)** Percent apoptotic nuclei in HEK cells expressing NIC1-GFP, NIC1-GFP + Sirt1, NIC1-GFP + Sirt1H363Y or GFP cultured with etoposide (10 µM) in medium containing 2% FBS for 48 h. HEK cells pre-treated with siRNA to NCL **(Bi)**, FBL **(Ci)**, or scrambled control for 48 h were transfected with indicated plasmids and cultured for 24 h before etoposide treatment. **(Bii,C-ii)** Immunoblots probed for Sirt1 and Tubulin in cell lysates from cells co-transfected with Sirt1, Sirt1H363Y or control pcDNA with NIC1-GFP and cultured for 48 h. Immunoblots are representative of three independent experiments. **(D)** Representative confocal images of HEK cells co-expressing NIC1-GFP, Fibrillarin RFP and Sirt1 or Sirt1H363Y imaged 24 h post-transfection. Images are representative of at least 40 cells across two independent experiments. 0.49 ± 0.09 (NIC1-GFP + Sirt1) and 0.24 ± 0.04 (NIC1-GFP + Sirt1H363Y) show the relative NIC1 levels in the nucleolus as compared to the nucleoplasm. Data plotted as mean ± S.D. of three independent experiments. *** and ** show significant difference with *p*-value ≤ 0.001 and ≤0.01, respectively, and ns shows non-significant difference examined using the unpaired student’s t-test.

Co-expression of Sirt1 with NIC1-GFP did not modulate inhibition of etoposide induced apoptosis (Figure 2A). However, siRNA mediated depletion of NCL or FBL, abrogated NIC1-GFP mediated inhibition of apoptosis in cells co-transfected with Sirt1, unlike cells expressing NIC1-GFP and pcDNA control vector (Figure 2C and Supplementary Figure S2A). To test if this required Sirt1 enzymatic activity, cells co-expressing NIC1-GFP and a catalytically inactive Sirt1 (Sirt1H363Y) recombinant were assessed in the assay of apoptosis. Co-expression of Sirt1H363Y, did not modulate NIC1-GFP mediated inhibition of apoptosis. Further, the ablation of nucleolar proteins did not change NIC1 mediated activity indicating that Sirt1 activity was most likely linked to its deacetylase activity (Figures 2Bi,Ci). Expression of Sirt1 was confirmed by western blotting and was increased in cells transfected with Sirt1 or SirtH363Y plasmids compared to cells transfected with control pcDNA (Figure 2C and Supplementary Figure S2B). These experiments suggest that Sirt1 mediated de-acetylation and nucleolar localization of NIC1 may induce dependence on nucleolar proteins. This possibility was strengthened by imaging analysis, where NIC1-GFP in cells co-expressing Sirt1 and FBL-RFP could be detected in the nucleolus (Figure 2D), as compared to NIC1-GFP distribution when co-expressed with Sirt1H363Y and FBL-RFP (Figure 2D).

Since Sirt1 activity was implicated in the deacetylation of lysine residues in NIC1, in the experiments that follow, the requirement of lysine residues (K1774, K1780, K1781 and K1782) for regulating NIC1 localization to the nucleolus was tested.

### Sirt1 targets lysine residues in NoLS to promote NIC1 localization in the nucleolus

**FIGURE 3:**
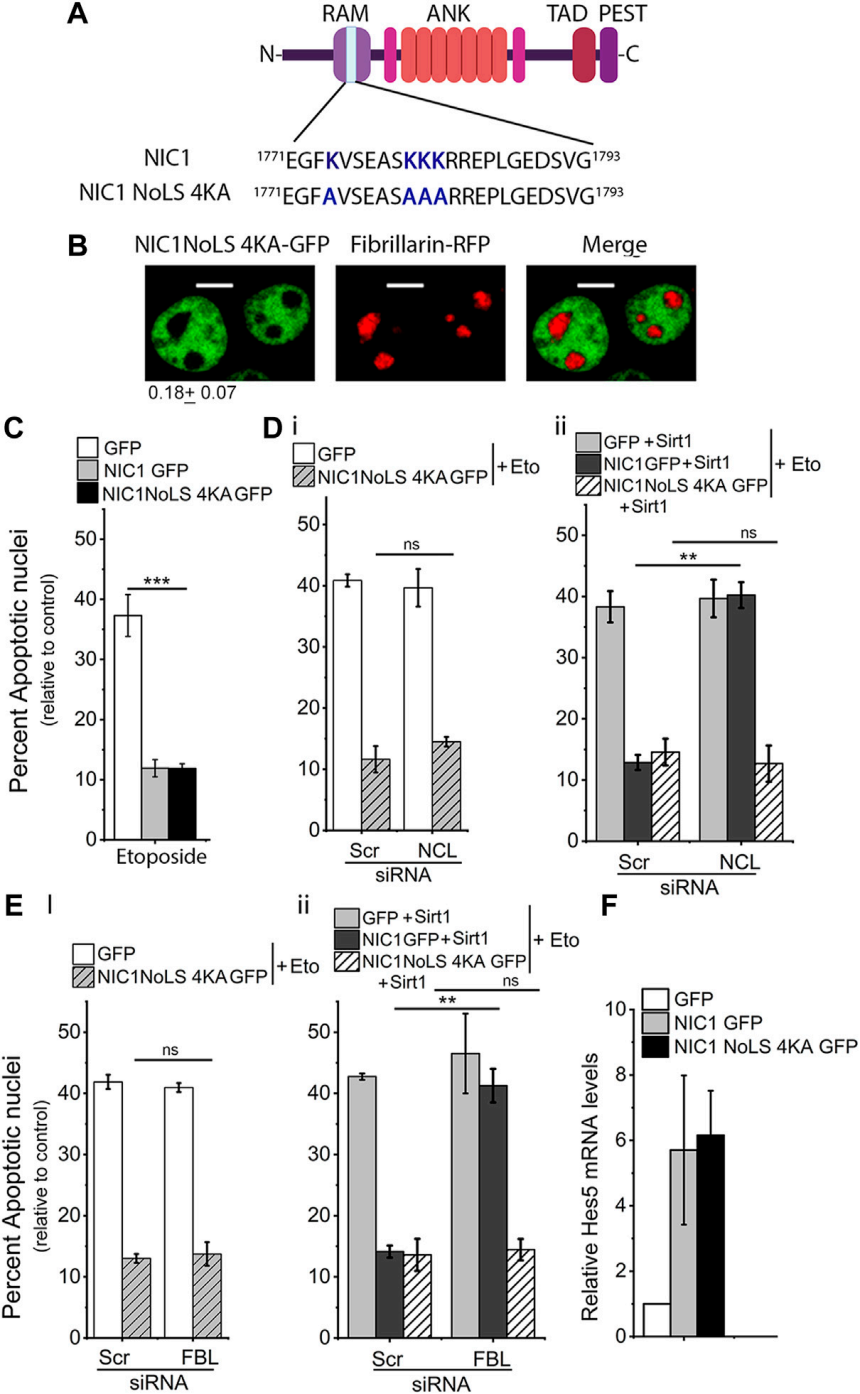
Lysine (K) to alanine **(A)** mutation in NIC1 abrogates dependence on FBL and NCL for protection against genomic damage. **(A)** Schematic showing lysine (K) to alanine (A) mutation in the putative NoLS in NIC1. **(B)** Representative confocal images of HEK cells co-transfected with NIC1-NoLS4KA-GFP and Fibrillarin-RFP and cultured for 24 h in complete medium post-transfection. Images are representative of 50 cells across two independent experiments. Scale bar: 5 μm. 0.18 ± 0.07 shows the relative NIC1-NoLS 4KA levels in the nucleolus as compared to the nucleoplasm. **(C)** Percent apoptotic nuclei in HEK cells expressing NIC1-NoLS 4KA-GFP, NIC1-GFP, or GFP cultured with etoposide (10 µM) for 48 h in medium containing 2% FBS. **(D,E)** Percent apoptotic nuclei in HEK cells expressing NIC1-NoLS 4KA-GFP or GFP **(Di,Ei)**, NIC1 GFP + Sirt1, NIC1-NoLS 4KA-GFP + Sirt1 or GFP + Sirt1 **(Dii,Eii)** cultured with etoposide (10 µM) in medium containing 2% FBS for 48 h. HEK cells pre-treated with siRNA to NCL **(D)**, FBL **(E)**, or scrambled control for 48 h were transfected with indicated plasmids and cultured for 24 h before etoposide treatment. **(F)** Induction of *Hes5* transcript levels in cells expressing NIC1-GFP or NIC1-NoLS 4KA-GFP relative to cells expressing the control GFP vector. Data plotted as mean ± S.D. of three independent experiments. *** and ** show significant difference with *p*-value ≤ 0.001 and ≤0.01, respectively, and ns shows non-significant difference examined using the unpaired student’s t-test.

The Lysine residues (K1774, K1780, K1781 and K1782) were modified by site-directed mutagenesis to alanine to generate the NIC1-NoLS 4KA recombinant (Figure 3A). Analysis of NIC1-NoLS 4KA-GFP localization in cells co-expressing FBL-RFP, showed that NIC1-NoLS 4KA-GFP is excluded from the nucleolus, marked by FBL-RFP (Figure 3B). Analysis of anti-apoptotic activity showed that over-expression of NIC1-NoLS 4KA-GFP protects cells against etoposide-induced damage (Figure 3C). Co-expression of Sirt1 and NIC1-NoLS 4KA-GFP protected cells from induction of apoptosis. However, siRNA mediated depletion of NCL (Supplementary Figure S3A) or FBL (Supplementary Figure S3B) did not change NIC1-NoLS 4KA-GFP mediated inhibition of etoposide induced apoptosis, even when co-expressed with Sirt1 (Figures 3D,E). The Sirt1 induced dependence on NCL or FBL for NIC1 mediated protection against genomic damage was observed as expected and formed the experimental control (Figures 3Dii,Eii). Induction of *Hes5* transcripts was comparable in cells expressing NIC1-NoLS 4KA-GFP or NIC1-GFP (Figure 3F). Together, these experiments suggested that Sirt1 modification of lysine residues in NoLS regulates NIC1 localization and dependence on NCL and FBL.

Mutation of the four Lysine residues in the NoLS to Alanine made NIC1 resistant to acetylation in NoLS and thus was independent of Sirt1 activity. However, Lysine to Alanine mutation also reduced the net positive charge in NoLS, essential for a functional NoLS (Scott and Oeffinger, 2016). Thus, NIC1-NoLS 4KA was not localized to the nucleolus. To further corroborate these observations, the localization and anti-apoptotic activity of NIC1 was tested in cells expressing a NIC1 mutant where the only modification was that lysine residues in the NoLS are mutated to arginine (R). Arginine cannot be modified by acetylation and hence mimics non-acetylated lysine.

### Modification of specific lysine residues regulates NIC1 localization in the nucleolus

**FIGURE 4:**
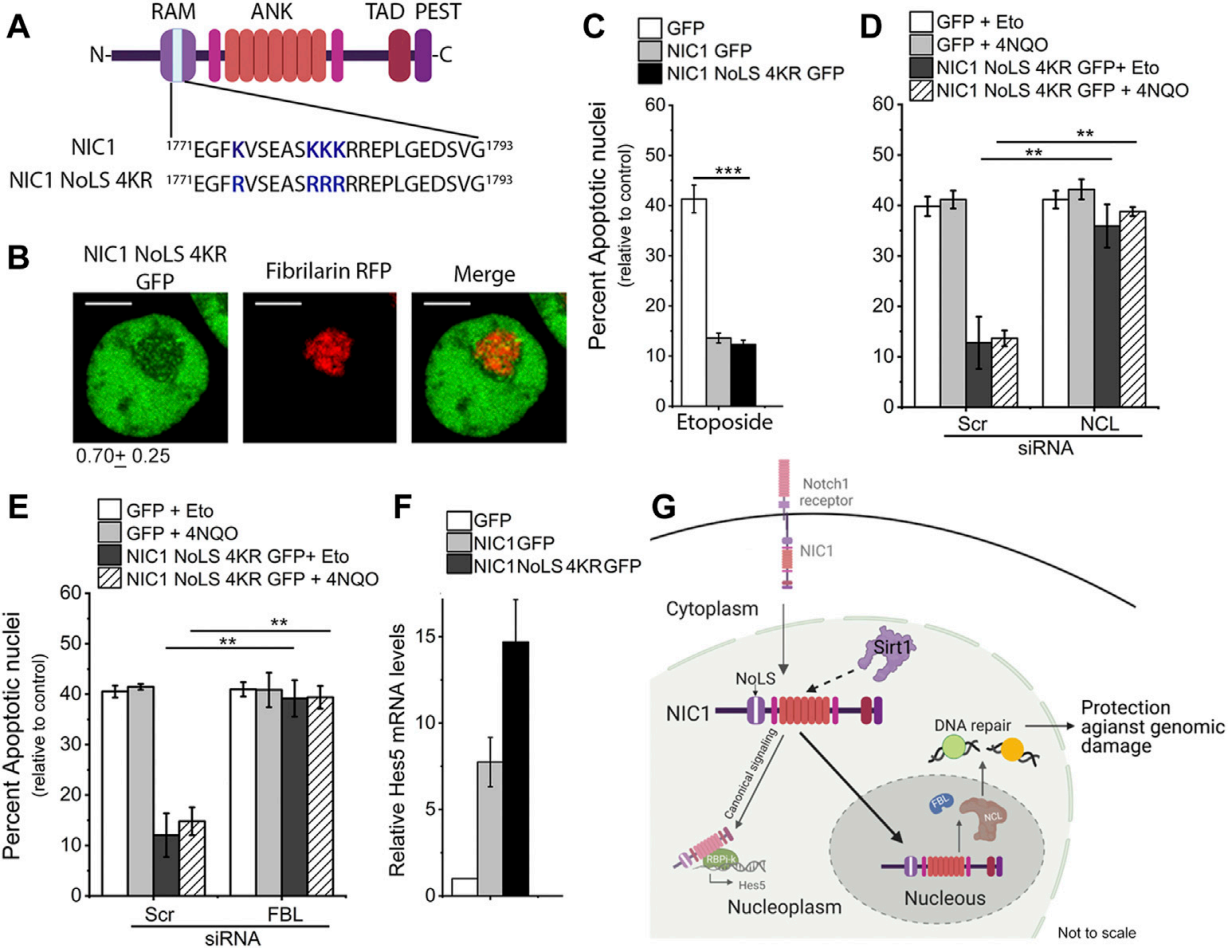
Lysine (K) to arginine (R) mutation in NIC1 promotes localization to the nucleolus. **(A)** Schematic showing lysine (K) residues mutated to arginine (R) in putative NoLS in NIC1. **(B)** Representative confocal images of HEK cells co-transfected with NIC1-NoLS 4KR-GFP and Fibrillarin-RFP and cultured for 24 h in complete medium before imaging. Images are representative of 50 cells across two independent experiments. Scale bar: 5 μm. 0.79 ± 0.25 shows the relative NIC1-NoLS 4KR levels in the nucleolus as compared to the nucleoplasm. **(C)** Percent apoptotic damage in HEK cells expressing NIC1-GFP, NIC1-NoLS 4KR-GFP or GFP treated with etoposide (10 µM) for 48 h in medium containing 2% FBS. **(D,E)** Percent apoptotic damage in HEK cells expressing NIC1-GFP, NIC1-NoLS 4KR-GFP or GFP treated with etoposide (10 µM) or 4NQO (10 µM) for 48 h in medium containing 2% FBS. HEK cells pre-treated with siRNA to NCL **(D)**, FBL **(E)**, or scrambled control were transfected with plasmids and cultured for 24 h before etoposide or 4NQO treatment. **(F)** Relative *Hes5* mRNA expression in HEK cells transfected with GFP, NIC1-GFP, or NIC1-NoLS 4KR-GFP and cultured for 30 h in the completed medium. **(G)** Schematic (not to scale) summarising the key outcomes. Sirt1 activity on lysine residues (K1774, K1780, K1781 and K1782) in putative NoLS promotes NIC1 localization to the nucleolus and induces dependence on nucleolar proteins for protection against genomic damage. Data plotted as mean ± S.D. of three independent experiments. *** and ** show significant difference with *p*-value ≤ 0.001 and ≤0.01, respectively, examined using the unpaired student’s t-test.

K1774, K1780, K1781, and K1782 residues were mutated to non-acetylatable, positively charged residue arginine (R) (Figure 4A). In cells co-transfected with NIC1-NoLS 4KR-GFP and FBL-RFP. NIC1-NoLS 4KR-GFP showed some overlap with FBL-RFP in the nucleolus (Figure 4B). Over-expression of NIC1-NoLS 4KR-GFP inhibited induction of apoptotic nuclei triggered by etoposide treatment to the same extent as observed with the expression of NIC1-GFP (Figure 4C). However, in contrast to NIC1, and in agreement with activity from the nucleolus, siRNA mediated depletion of either NCL or FBL abrogated NIC1-NoLS 4KR-GFP mediated inhibition of apoptosis (Figures 4D,E and Supplementary Figures S4A,B). Cells expressing NIC1-NoLS 4KR-GFP showed an increase in *Hes5* transcript levels, relative to that of NIC1-GFP expressing cells (Figure 4F).

Taken together, the data suggest that acetylation of lysine residues (K1774, K1780, K1781 and K1782) in the putative NoLS in NIC1 prevents its localization to the nucleolus. Intriguingly, whilst not dependent on nucleolar localization for anti-apoptotic activity, nucleolar localized NIC1 can interact with nucleolar proteins–NCL and FBL—to activate an anti-apoptotic cascade. The experiments also provide evidence that Sirt1 modification of the lysine residues in NIC1-NoLS is sufficient to trigger this switch.

## Discussion

Reversible post-translational modifications of signaling molecules specifically, acetylation are shown to regulate a number of signaling pathways including Notch signaling (Borggrefe et al., 2016; Bai et al., 2018; Collesi et al., 2018). Acetylation of lysine residues in NIC regulates localization and stability and thereby signaling outcomes (Guarani et al., 2011; Marcel et al., 2017). The understanding of distinct functional outcomes as a result of acetylation of different lysine residues in NIC1 is still lacking. Here we provide evidence that posttranslational modification of the lysine residues, K1774, K1780, K1781, and K1782 in the RAM domain of NIC1 regulates its localization to the nucleolus and that Sirtuin1 can regulate this outcome. We also show that NIC1 signaling from the nucleolus integrates with nucleolar proteins, Nucleolin and Fibrillarin, to inhibit apoptosis triggered by genotoxic stressors (Figure 4G). Transcriptional functions of NIC1 were not so regulated.

Non-canonical, NIC1 signaling from the nucleolus inhibits apoptosis induced by genomic damage (Vermezovic et al., 2015). NIC1 competes with FOXO3a and interacts with ATM and thereby blocking the activation of ATM and downstream apoptotic cascades (Adamowicz et al., 2016). Consistent with these studies, NIC1 signaling from the nucleoplasm was observed to protect cells against genomic damage. Intriguingly, NIC1 signaling from the nucleolus, activated a distinct signaling cascade requiring nucleolar proteins FBL and NCL to promote cell survival. Deletion of TAD PEST domains enabled NIC1 localization in the nucleolus and induced dependence on nucleolar proteins indicating the intramolecular regulation of NIC1 localization and anti-apoptotic activity. Lysine to alanine nutation in NIC1-ΔTADPEST-GFP blocked localization in the nucleolus and analysis of NIC1 mediated anti-apoptotic activity against genomic damage suggested that NIC1-ΔTAD PEST localized in the nucleolus, accounts for *all* the protection conferred from genomic damage. It may be noted that transcriptional activity of NIC1-ΔTADPEST-GFP is also attenuated suggested a loss of NIC1 function in the nucleoplasm (Aster et al., 2000; Kurooka and Honjo, 2000; Kopan and Ilagan 2009).

Sirtuin1 modification of Notch1 or mimicking Sirtuin1 modification of the 4 Lysine residues resulted in the functional dominance of the nucleolar pathway of anti-apoptotic activity activated by Notch1. At this point, we don’t fully understand, how the dominance of the nucleolar anti-apoptotic pathway is established for protection against genomic damage. Post-translation modifications (acetylation and sumoylation) of lysine residues in NoLS are shown to tune Notch1 functions by modulating protein-protein interactions or stability (Guarani et al., 2011; Antila et al., 2018). Thus, one possibility is that change in the post-translation modification of these residues is altering protein stability or interaction with other proteins resulting in the loss of anti-apoptotic activity from the nucleoplasm. The nucleolus is primarily implicated in ribosome biogenesis and the DNA repair process (Scott and Oeffinger, 2016; Lindström et al., 2018). Nucleolar proteins NCL and Nucleophosmin (NPM) participate in DNA damage response and promote DNA repair (Kobayashi et al., 2012; Goldstein et al., 2013; Poletto et al., 2014). Co-expression of the different NIC1 constructs used in the study did not change FBL-RFP distribution in the cells. Data presented in the study showed that in contexts when NIC1 appears to be localized to the nucleolus, there is a corresponding dependence on NCL or FBL for protection from genomic damage. However, whether this functional interaction between NIC1 and FBL and NCL proteins is direct or only restricted to the nucleolus has not been addressed in the present study and requires further investigation.

Immunostaining of Notch1 in mouse and rabbit trophoblast stem cells showed that NIC1 is enriched in the nucleolus (Sarikaya and Jerome-Majewska, 2011; Tan et al., 2014). This is in line with the observation that Sirtuin1 is highly expressed in embryonic stem cells as compared to differentiated cells and promotes DNA repair and enhances the survival of human embryonic stem cells (Han et al., 2008; Jang et al., 2017). Efficient DNA repair is critical for genomic stability and the transfer of correct genetic information, which can be particularly critical for stem cells. Human and mouse embryonic stem cells have been shown to be hypersensitive to DNA damage and show enhanced DNA repair activity as compared to differentiated counterparts (Maynard et al., 2008; Liedtke et al., 2015).

In summary, this report delineates NIC1 signaling from the nucleolus in the protection against genomic damage and position Sirt1 modification underpinning NIC1 localization in the nucleolus. These data add to the evidence of the remarkable versatility of Notch1 signaling, wherein intra-molecular interactions suffice to enable signaling from distinct cellular locations, reported here in the context of apoptosis induced by genomic damage (Harvey and Haltiwanger, 2018, Mungamuri et al., 2006).

## Data Availability

The original contributions presented in the study are included in the article/Supplementary Material, further inquiries can be directed to the corresponding author.

## Abbreviations

FBL, Fibrillarin; Hes5, Hairy/Enhancer of Split 5; NIC1, Notch1 intracellular domain; NIC4, Notch4 intracellular domain; NCL, Nucleolin; NoLS, Nucleolar Localization Sequence; 4NQO, 4-Nitroquinoline 1-Oxide; Sirt1, Sirtuin1.
