# Supplementary material for: Sirtuin1 meditated modification of Notch1 intracellular domain regulates nucleolar localization and activation of distinct signaling cascades
Source: Front Cell Dev Biol. 2022 Sep 23;10:988816. doi: 10.3389/fcell.2022.988816 (PMC9539544; doi:10.3389/fcell.2022.988816)
Supplement: Supplementary file 1 [file Image1.pdf]

Supplementary Material

Supplementary Figure 1

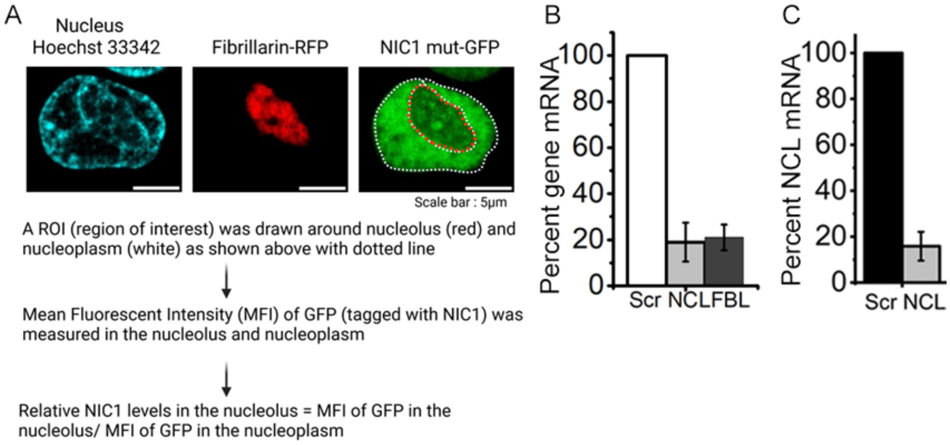

Supplementary figure 1: (A) Schematic showing the measurement of relative NIC1 levels in nucleolus. (B) Percent transcript levels of indicated genes in cells treated with siRNA to FBL, NCL and scrambled control. (C) Percent transcript levels of NCL in cells treated with siRNA to NCL and scrambled control. Data plotted as mean ± S.D. of three independent experiments.

Supplementary Figure 2

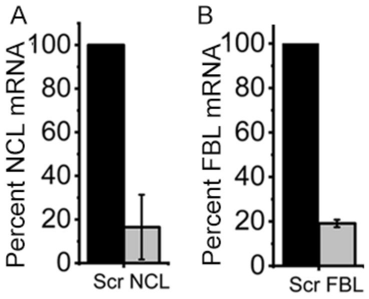

Supplementary figure 2: (A) Percent transcript levels of NCL in cells treated with siRNA to NCL and scrambled control. (B) Percent mRNA levels of FBL in cells treated with siRNA to FBL and scrambled control. Data plotted as mean ± S.D. of three independent experiments.

Supplementary Figure 3

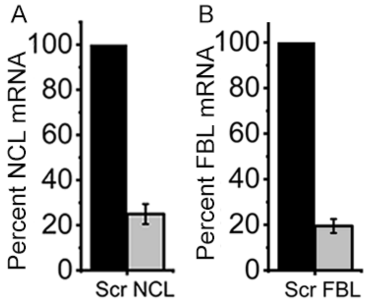

Supplementary figure 3: (A) Percent mRNA levels of NCL in cells treated with siRNA to NCL and scrambled control. (B) Percent transcript levels of FBL in cells treated with siRNA to FBL and scrambled control. Data plotted as mean ± S.D. of three independent experiments.

Supplementary Figure 4

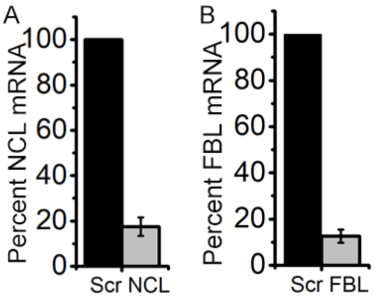

Supplementary figure 4: (A) Percent transcript levels of NCL in cells treated with siRNA to NCL and scrambled control. (B) Percent mRNA levels of FBL in cells treated with siRNA to FBL and scrambled control. Data plotted as mean ± S.D. of three independent experiments.
